# Relationships between heavy metal concentrations in three different body fluids and male reproductive parameters: a pilot study

**DOI:** 10.1186/1476-069X-10-6

**Published:** 2011-01-19

**Authors:** Jaime Mendiola, José M Moreno, Manuela Roca, Nuria Vergara-Juárez, María J Martínez-García, Antonio García-Sánchez, Belén Elvira-Rendueles, Stella Moreno-Grau, José J López-Espín, Jorge Ten, Rafael Bernabeu, Alberto M Torres-Cantero

**Affiliations:** 1 Public Health and Epidemiology Research Group, Division of Preventive Medicine and Public Health, School of Medicine, University of Murcia, Espinardo (Murcia), Spain; 2 Department of Environmental and Chemical Engineering, Technical University of Cartagena, Cartagena, Spain; 3 Department of Statistics, Mathematics and Informatics, Miguel Hernández University, Elche, Spain; 4 Department of Reproductive Biology and Medicine, Instituto Bernabeu, Alicante, Spain; 5 Reproductive Medicine Chair, Miguel Hernández University-Instituto Bernabeu, Alicante, Spain; 6 Centro de Investigación Biomédica en Red de Epidemiología y Salud Pública (CIBERESP), Barcelona, Spain

## Abstract

**Background:**

Animal studies have shown the reproductive toxicity of a number of heavy metals. Very few human observational studies have analyzed the relationship between male reproductive function and heavy metal concentrations in diverse biological fluids.

**Methods:**

The current study assessed the associations between seminal and hormonal parameters and the concentration of the 3 most frequent heavy metal toxicants (lead, cadmium and mercury) in three different body fluids. Sixty one men attending infertility clinics that participated in a case-control study to explore the role of environmental toxins and lifestyles on male infertility were analyzed. Concentration of lead, cadmium and mercury were measured in blood and seminal plasma and whole blood using anodic stripping voltammetry and atomic absorption spectrophotometry. Serum samples were analyzed for follicle-stimulating hormone, luteinizing hormone and testosterone. Semen analyses were performed according to World Health Organization criteria. Mann-Whitney test and Spearman's rank correlations were used for unadjusted analyses. Multiple linear regression models were performed controlling for age, body mass index and number of cigarettes per day.

**Results:**

There were no significant differences between cases and controls in the concentrations of heavy metals in any of the three body fluids. In multivariate analyses using all subjects no significant associations were found between serum hormone levels and metal concentrations. However there was a significant positive association between the percentage of immotile sperms and seminal plasma levels of lead and cadmium.

**Conclusions:**

Our results suggest that the presence of lead and cadmium in the reproductive tract of men may be related to a moderate alteration of their seminal parameters.

## Background

Human, animal and in vitro studies suggest that heavy metals may have adverse impacts on male reproductive health [1-10], even at relatively low-level exposures [11]. Heavy metals could adversely affect the male reproductive system, either by causing hypothalamic-pituitary axis disruption or by directly affecting spermatogenesis, resulting in impair semen quality [12].

Several metals - mainly lead (Pb), cadmium (Cd) - are considered reproductive toxicants and/or suspected endocrine disruptor compounds. Human populations could be exposed to heavy metals at trace concentrations usually through intake of contaminated water and food or contact with contaminated air or soil.

A number of studies have reported a significant inverse association between blood and seminal Pb concentrations and semen quality among both occupationally exposed and unexposed men [4,5,10,13]. Cd has been related to impaired semen quality and altered hormonal levels in men [9,10,14-17]. Cd is considered as an endocrine disruptor, but the mechanisms involved are still unclear [18]. Animal and in vitro studies have shown that mercury (Hg) can induce abnormalities in sperm morphology and motility [19,20]. Choy et al. [2] described an association between semen Hg concentrations and sperm abnormalities in subfertile males attending an infertility clinic. However, Meeker et al. [7] found no relationship between semen quality and Hg levels in blood.

The aim of this preliminary study was to explore the relationship between the most ubiquitous heavy metal toxicants (Pb, Cd and Hg) in three different body fluids and seminal and hormonal parameters among men attending infertility clinics.

## Methods

### Study population, design and semen analysis

The study population, hormonal and seminal analyses have been described in previous articles [21,22]. All men were participating in a study to explore the role of environmental toxins and lifestyles on male infertility. Briefly, the men of couples attending the three infertility centers of the Instituto Bernabeu in Murcia and Alicante (southeastern Spain) between 2005 and 2007 were classified into two groups on the basis of semen quality following World Health Organization (WHO) criteria [23]: 1) case subjects (n = 30) composed of men with oligo-astheno-teratozoospermia, and 2) control subjects (n = 31) composed of normospermic patients. Subjects provided two semen samples and were requested to observe a 3- to 5-day abstinence period. The importance of the abstinence period was stressed on the interviews with the participants [21]. The average of the two samples was used in our statistical analysis (Table 1). Semen parameters evaluated included: ejaculate volume, sperm concentration, percentage of motile and immotile sperm, and percentage of normal forms following Kruger's strict criteria [23]. All patients were interviewed face-to-face by the same interviewer and completed a comprehensive occupational and lifestyle questionnaire [21]. This study was approved by the Institutional Review Board of our clinics, and patients were included in the study after giving informed written consent.

**Table 1:** Characteristics of participants in the study.

	Case subjects (n = 30)	Control subjects (n = 31)	
	
	Mean (±SD)	Mean (±SD)	P value
Education			

% Low	30%	26%	NS

% Medium	56%	55%	NS

% High	14%	19%	NS

Alcohol consumption (drinks^1^/week)	6.5 (2.4)	6.8 (2.7)	NS

Smoking (cigarettes/day)	18.6 (15.6)	13.2 (2.9)	NS

Semen parameters^2^			

Seminal volume (mL)	3.8 (1.2)	3.5 (1.4)	

Sperm count (10^6^/mL)	3.3 (4.1)	39.5 (14.6)	

% Motile sperm (A+B)	27.4 (18.6)	52.2 (12.3)	

% Normal morphology	3.7 (1.5)	22.3 (4.5)	

Total sperm count (10^6^)	12.6 (9.1)	151 (71.9)	

SD: standard deviation

NS: not significant

^1^One drink : 33 cL beer, 10 cL wine or 33 cL liquor.

^2^Average of two samples; no significant differences were found between the first and second samples within cases or controls.

### Hormone measurement

Blood samples were withdrawn from a cubital vein of each participant, centrifuged and the serum was stored at -20 °C until analysis. Follicle-stimulating hormone (FSH), luteinizing hormone (LH) and testosterone (T) levels were measured by enzyme linked fluorescent assay (ELFA) using the BioMérieux VIDAS Automated Immunoassay System (Biomérieux^® ^S.A., Marcy-l'Etoile, France). Intra- and inter-assay coefficients of variation for all hormones were below 10%. Assay sensitivities were 0.1 IU/L for FSH and LH and 0.08 ng/mL for T.

### Measurements of metals

A total of 181 biological samples were analyzed for Pb, Cd and Hg, including 61 samples of seminal plasma, 61 of blood plasma and 59 of whole blood, as two samples were lost during the study. Biological samples were dispensed into aliquots and frozen and stored at -40°C until analysis. Anodic Stripping Voltammetry (ASV) was used for measuring Pb and Cd concentrations. ASV was carried out using a voltamperometer with VA 663 stand and VA 608 controller (Metrohm 626, Herisau, Switzerland). The voltamperometric cell was equipped with a drop of mercury as the working electrode, an Ag/AgCl/KCl 3 M reference electrode and a platinum auxiliary electrode.

Determination of total Hg was carried out by thermal decomposition, amalgamation and atomic absorption spectrophotometry, using a Mercury analyzer with quartz sample boats (DMA-80 Direct Mercury Analyzer, Milestone, Shelton CT, USA).

The highest grade purity reagents were employed in this procedure including nitric acid 65% and perchloric acid 70% (Suprapur^®^, Merck, Darmstadt, Germany). The double-distilled water was purified with Millipore Simplicity 185 (Millipore GmbH, Molsheim, France) obtaining conductivity values of 0.054 μS/cm.

In order to prepare the working standard solutions, commercially available standard solutions for Pb 1 g/L and Cd 1 g/L (Tritisol^®^, Merck, Darmstadt, Germany) and Hg 1 g/L (Certipur^®^, Merck, Darmstadt, Germany) were used. The limits of detection (LOD) for the body's fluid metal levels were as follows: lead, 21 μg/L; cadmium, 0.11 μg/L and mercury, 0.1 μg/L. To guarantee the accuracy and precision of the applied technique regarding heavy metals, whole blood reference materials (Seronorm™ Trace Elements Whole Blood, SERO AS, Billingstad, Norway) were employed.

### Sample preparation

Pb and Cd determinations were performed using 0.2 mL of the biological sample deposited inside of 25 mL borosilicate glass. Acid digestion was carried out by adding 2 mL of nitric acid and 2 mL of perchloric acid and evaporating it to dryness. Once the sample was dry and cooled down, 100 μl of perchloric acid and 15 mL of double-distilled water were added, transferring the final volume into a voltamperometric cell.

Biological samples were measured by ASV according to the following method [24]. Briefly, differential pulse (DP) with hanging mercury drop electrode (HMDE) was used, the voltage sweep was from -0.70 to +0.15 volts and the peak voltage was located at -0.58 and -0.40 volts for Cd and Pb respectively. Deaeration, preconcentration and resting time (without stirring) were 180, 120 and 40 seconds respectively. Sensitivity was 0.05 nAmp/mm and 0.2 nAmp/mm for Cd and Pb respectively.

Standard addition method was applied to perform the current analyses, adding known values of a standard solution (2, 4 and 6 ng for Cd and 20, 40 and 60 ng for Pb) to obtain a calibration curve, then the values of the measurements were interpolated into that curve.

Mercury determination was carried out following EPA method 7473 [25] and 0.2 mL of the biological sample was transferred directly into the quartz sample boats. To obtain a calibration curve, standard solutions of 5, 10, 20, 30, 100, 200 and 500 ng of Hg were employed.

### Statistical analysis

Statistical analysis was performed with the statistical package SPSS 17.0 (SPSS Inc., Chicago, IL, USA). Descriptive statistics are presented using untransformed data. In preliminary analyses (unadjusted), Mann-Whitney test was used to explore the relationship between cases and control subjects. In order to assess the relationship between each hormone level and sperm parameter and each heavy metal concentration in the entire population Spearman's rank correlations were employed. Heavy metal concentrations and sperm concentration showed skewed (non-normal) distributions and were natural log (ln) transformed. Multiple linear regression analysis was then performed controlling for appropriate covariates, including age, body mass index (BMI) and number of cigarettes per day. All covariates were modeled as continuous variables. All tests were two-tailed and the level of statistical significance was set at 0.05.

## Results

Table 1 shows characteristics of the participants in the study. The overall mean age [in years ± standard deviation (SD)] and BMI (in kg/m^2^ ± SD) were 33.5 ± 3.8 and 23.2 ± 2.5, respectively. Thirty-one percent of the men were smokers and all were white Caucasian. Summary statistics for serum hormone levels and heavy metal concentrations in seminal and blood plasma and whole blood are shown in Table 2. All the samples were above the LOD. None of the three body fluids showed significant differences between case and control subjects regarding heavy metal concentrations (Table 3). Because no differences were found between the two groups, we explored the relationship between each hormone level and sperm parameter and each metal concentration in the entire population. Table 4 shows Spearman's rank correlation coefficients between metal concentrations in seminal and blood plasma and whole blood. There was no correlation between concentrations of each individual metal in any of the three body fluids. Nor were seminal plasma, blood plasma or whole blood levels correlated for Pb, Cd and Hg independently. However, the concentrations of the 3 metals (Pb, Cd and Hg) in each body fluid were highly correlated when compared in the same body fluid. No significant associations were found between serum hormone levels and metal concentrations (Table 5). Finally, there was a significant positive correlation between the percentage of immotile sperms and seminal plasma levels of Pb and Cd. That association remained statistically significant after adjustment for age, BMI and number of cigarettes per day (β= 1.5; 95% CI, 0.37, 1.9 and β= 4.9; 95% CI, 0.84, 9.1, respectively) (Table 6). An inter-quartile increase in seminal plasma concentrations of Pb (5.0 μg/L) and Cd (0.3 μg/L) for a 33.5-year-old with BMI of 23 kg/m^2 ^would be predicted to increase immotile sperms 21.6% and 24.3%, respectively.

**Table 2:** Summary statistics for plasma hormone levels and metal concentrations in seminal and blood plasma (n = 61 samples each) and whole blood (n = 59 samples).

	Geometric Mean	Selected Percentiles			
		
		**25**^**th**^	**50**^**th**^	**75**^**th**^	**95**^**th**^
Variables					

FSH (IU/L)	6.1	5.0	7.0	8.0	9.0

LH (IU/L)	3.9	3.2	4.2	5.2	6.1

Testosterone (ng/mL)	5.1	4.6	5.1	6.2	8.4

Lead (μg/L)					

Seminal plasma	29.0	27.0	29.0	32.0	36.0

Blood plasma	29.0	27.0	29.0	31.0	33.0

Whole blood	95.0	75.0	101	119	133

Cadmium (μg/L)					

Seminal plasma	0.8	0.7	0.8	1.0	1.1

Blood plasma	0.8	0.75	0.8	0.84	0.9

Whole blood	1.0	0.9	1.0	1.2	1.5

Mercury (μg/L)					

Seminal plasma	11.3	9.2	11.3	14.9	18.0

Blood plasma	5.7	4.2	5.8	7.2	10.6

Whole blood	18.8	14.7	19.6	24.6	33.1

**Table 3:** Heavy metal concentrations in seminal and blood plasma and whole blood among case and control subjects.

	Case subjects (n = 30)	Control subjects (n = 31)	
	
	Mean (±SD)	Mean (±SD)	P value
Lead S.P. (μg/L)	30 (3.0)	29 (3.4)	NS

Lead B.P. (μg/L)	29 (2.0)	29 (2.5)	NS

Lead W.B. (μg/L)^1^	98 (23)	97 (23)	NS

Cadmium S.P. (μg/L)	0.9 (0.1)	0.8 (0.1)	NS

Cadmium B.P. (μg/L)	0.7 (0.1)	0.8 (0.1)	NS

Cadmium W.B. (μg/L)^1^	1.1 (0.2)	1.1 (0.2)	NS

Mercury S.P. (μg/L)	12.3 (3.7)	11.5 (3.4)	NS

Mercury B.P. (μg/L)	5.8 (2.4)	6.2 (2.0)	NS

Mercury W.B. (μg/L)^1^	19.9 (6.8)	20.0 (7.3)	NS

S.P.: Seminal plasma

B.P.: Blood plasma

W.B.: Whole blood

SD: standard deviation

NS: not significant

^1^n = 59

**Table 4:** Spermean's correlation coefficients between metal concentrations in seminal and blood plasma (n = 61 samples each) and whole blood (n = 59 samples).

	Lead S.P. R (P)	Lead B.P. R (P)	Lead W.B. R (P)	Cadmium S.P. R (P)	Cadmium B.P. R (P)	Cadmium W.B. R (P)	Mercury S.P. R (P)	Mercury B.P. R (P)
Lead B.P.	.13 (.32)							

Lead W.B.	-.08 (.55)	.06 (.67)						

Cadmium S.P.	.**79 (.001)**	.14 (.29)	-.06 (.66)					

Cadmium B.P.	.18 (.17)	**.60 (.001)**	.08 (.54)	.12 (.36)				

Cadmium W.B.	.01 (.97)	-.02 (.86)	.**88 (.001)**	-.05 (.71)	.14 (.29)			

Mercury S.P.	.**79 (.001)**	.04 (.79)	-.10 (.48)	.**88 (.001)**	.08 (.56)	-.08 (.55)		

Mercury B.P.	-.06 (.63)	.**75 (.001)**	.15 (.25)	-.05 (.70)	.**71 (.001)**	.22 (.09)	-.13 (.33)	

Mercury W.B.	-.10 (.47)	.11 (.42)	.**95 (.001)**	-.01 (.92)	.10 (.47)	**.82 (.001)**	-.03 (.80)	.17 (.19)

S.P.: Seminal plasma

B.P.: Blood plasma

W.B.: Whole blood

R = Spermean's rank correlation

P = P value

**Table 5:** Multivariate analysis for male reproductive hormones and metal concentrations in seminal and blood plasma and whole blood^1^.

	FSH		LH		T	
	
	β	95% CI	β	95% CI	β	95% CI
Lead S.P.	.05	(-.24, .39)	.14	(-.13, .41)	.11	(-.10, .31)

Lead B.P.	-.20	(-.64, .25)	-.07	(-.49, .31)	-.12	(-.40, .14)

Lead W.B.	.04	(-.03, .04)	.05	(-.05, .07)	.01	(-.05, .02)

Cadmium S.P.	-.02	(-.03, .03)	3.7	(-3.1, 10.3)	4.3	(-.55, 10.1)

Cadmium B.P.	-1.9	(-9.3, 5.9)	-4.3	(-18.3, 8.9)	-9.2	(-22.7, 2.1)

Cadmium W.B.	-12.0	(-25.0, 3.9)	4.4	(-.41, 9.5)	-.22	(-3.3, 3.5)

Mercury S.P.	.06	(-.02, .05)	.01	(-.01, .06)	.03	(-.01, .04)

Mercury B.P.	-.07	(-.08, .02)	-.03	(-.08, .02)	-.02	(-.05, .02)

Mercury W.B.	.02	(-.07, .01)	.02	(-.01, .02)	.003	(-.01, .02)

^1^Controlling for age, BMI and number of cigarettes per day

S.P.: Seminal plasma

B.P.: Blood plasma

W.B.: Whole blood

β= regression coefficient

CI = confidence interval

Heavy metal concentrations were ln-transformed

**Table 6:** Multivariate analysis for semen quality parameters and metal concentrations in seminal and blood plasma and whole blood^1^.

	Sperm concentration 10^6^/mL		% Immotile Sperm		% Morphologically Normal Sperm	
	
	β	95% CI	β	95% CI	β	95% CI
Lead S.P.	-1.0	(-3.1, 2.3)	1.5	(.37, 1.9)^a^	-.54	(-3.1, 2.0)

Lead B.P.	.08	(-4.1, 5.2)	-.49	(-1.8, .62)	-.08	(-3.5, 3.4)

Lead W.B.	-.02	(-1.7, 1.6)	.05	(-.32, .43)	-.31	(-1.5, .89)

Cadmium S.P.	-7.2	(-20.3, 9.5)	4.9	(.84, 9.1)^a^	-3.5	(-15.8, 8.7)

Cadmium B.P.	3.1	(-26.2, 26.2)	-3.7	(-11.0, 4.3)	.-2.7	(-24.4, 19.1)

Cadmium W.B.	2.1	(-10.1, 16.9)	-.85	(-4.3, 2.6)	-.87	(-10.9, 9.1)

Mercury S.P.	-.06	(-.11, .11)	.03	(-.01, .05)	-.11	(-.91, .68)

Mercury B.P.	.07	(-.07, .20)	-.05	(-.09, .01)	-.01	(-1.3, 1.2)

Mercury W.B.	.04	(-.02, .09)	-.005	(-.02, .01)	.05	(-.37, .46)

^1^Controlling for age, BMI and number of cigarettes per day

^a^P value≤0.05

S.P.: Seminal plasma

B.P.: Blood plasma

W.B.: Whole blood

β = regression coefficient

CI = confidence interval

Heavy metal and sperm concentrations were ln-transformed

## Discussion

To our knowledge, this is the first study to examine the relationships between selected heavy metal concentrations (Pb, Cd and Hg) in three different male body fluids and seminal and hormonal parameters. We observed a significant positive association between the percentage of immotile sperms and seminal plasma levels of Pb and Cd after controlling for appropriate covariates. No significant associations were found between any heavy metal concentrations and sperm concentration or morphology.

Interestingly enough, the concentration of each metal was not correlated in any of the three body fluids. That finding is consistent with previously reported data [6,15]. Benoff et al. [15] did not find any correlations between Cd concentrations in seminal plasma and blood plasma in three populations of men that were studied. Similarly, Hernandez-Ochoa et al. [6] found no correlation between Pb concentrations in blood and seminal plasma, blood and spermatozoa, and seminal plasma and spermatozoa in 68 men residing in Region Lagunera (Mexico).

Nevertheless, the 3 heavy metals (Pb, Cd and Hg) were highly correlated among themselves when compared within the same body fluid. Therefore a higher level of a given metal in a body fluid indicates a higher concentration of the other metals in our study sample.

We found no significant associations between serum hormone levels and heavy metal concentrations in any of the 3 body fluids. A number of studies have investigated the association between exposure to heavy metals and hormone levels among occupationally exposed men, but results are controversial [26-28]. Among men with no occupational exposure, Jurasovic et al. [5] have reported positive associations between blood Cd concentrations and FSH and testosterone levels.

Several studies have reported declines in semen quality associated with both Pb [4,11,13] and Cd concentrations in blood [10,14]. However, we observed no significant associations between blood levels of heavy metals and any semen parameters.

On the other hand, and consistent with our findings, several reports have shown an association between impaired sperm motility and Cd and/or Pb concentrations in sperm or seminal fluid [3,6,9,15].

We compared the distribution of concentration of heavy metals in our study with other distributions already published that included only men with no occupational exposure. Our median blood and semen Pb concentrations (10.1 μg/dL and 2.9 μg/dL respectively) were similar to those published by Hernandez-Ochoa et al. [6] in Mexico or Jurasovic et al. [5] in Croatia (9.3 μg/dL and 5.7 μg/dL, respectively), but quite different, ten times higher, than the median blood Pb concentration (1.5 μg/dL) reported by Meeker et al. [8] in the US. Nevertheless, there are limitations when attempting to compare absolute concentration levels among the studies due to possible differences in methodology and laboratory techniques when measuring heavy metals.

Our results, in agreement with previous published studies, suggest that sperm motility might be one of the most sensitive parameters to be altered by Pb and Cd exposure [3,6,9,15,16]. Moreover, our results also suggest that Pb, Cd and Hg concentrations in the reproductive tract are not reflected by blood levels and those metals would be better assessed in seminal plasma [6,15,29].

Our data were limited by the use of a single blood sample to assess serum hormone levels and heavy metal concentrations. However, a single sample can be used to classify men's reproductive hormones [30] and metal exposures over several months [31]. Our study was also limited by selecting only men attending fertility clinics. Finally, as the number of participants was small, the study may be subjected to type II error and may not have had power enough to detect small associations. Larger studies involving other populations would be desirable.

## Conclusions

Our results of this preliminary study suggest that the presence of Pb and Cd in the reproductive tract of men attending infertility clinics may be related to a moderate alteration of their seminal parameters. Furthermore, blood concentrations of heavy metals do not reflect adequately their concentration in the male reproductive tract.

## List of Abbreviations

ASV: anodic stripping voltammetry; BMI: body mass index; Cd: cadmium; ELFA: enzyme linked fluorescent assay; EPA: Environmental Protection Agency; FSH: follicle-stimulating hormone; Hg: mercury; HMDE: hanging mercury drop electrode; LH: luteinizing hormone; LOD: limits of detection; Pb: lead; SD: standard deviation; T: testosterone; WHO: World Health Organization.

## Competing interests

The authors declare that they have no competing interests.

## Authors' contributions

MR, JM, RB and AMT-C designed and initiated the current study. MR, JM, JT and RB were responsible for collecting the samples and the interview data. JMM, NV-J, MJM-G, BE-R and SM-G were responsible for the chemical analysis. JM and MR coordinated the current study. JJL-E and AG-S were responsible for statistical analysis. JM, MR, JMM, SM-G and AMT-C were responsible for writing the draft version of manuscript. All authors commented on and approved the final manuscript.
